# Supplementary material for: MetaRibo-Seq measures translation in microbiomes
Source: Nat Commun. 2020 Jun 29;11:3268. doi: 10.1038/s41467-020-17081-z (PMC7324362; doi:10.1038/s41467-020-17081-z)
Supplement: Supplementary file 10 — Supplementary Data 7 [file 41467_2020_17081_MOESM10_ESM.zip › File2/Confidence_High_Taxonomy/89213_out.krona.html]

Javascript must be enabled to view this page.

members
magnitude
magnitudeUnassigned
count
unassigned
taxon
rank

89213\_out

4

4
superkingdom
2

1239
4
phylum

186801
class
4

order
4
186802

2109688

SRS017821\_contig\_number\_26639SRS020869\_contig\_number\_38485SRS1041118\_contig\_number\_16428SRS893373\_contig\_number\_7654
4
species
